# Endoglin potentiates nitric oxide synthesis to enhance definitive hematopoiesis

**DOI:** 10.1242/bio.011494

**Published:** 2015-05-15

**Authors:** Rabab Nasrallah, Kathy Knezevic, Thuan Thai, Shane R. Thomas, Berthold Göttgens, Georges Lacaud, Valerie Kouskoff, John E. Pimanda

**Affiliations:** 1 Lowy Cancer Research Centre and the Prince of Wales Clinical School, UNSW Australia, Sydney, NSW 2052, Australia; 2 Cancer Research UK Manchester Institute, The University of Manchester, Manchester, M20 4BX, UK; 3 Centre for Vascular Research and School of Medical Sciences, UNSW Australia, Sydney, NSW 2052, Australia; 4 Department of Haematology, Cambridge Institute for Medical Research, University of Cambridge, Cambridge CB2 0XY, UK; 5 Cambridge Stem Cell Institute, University of Cambridge, Cambridge CB2 1QR, UK; 6 Department of Haematology, Prince of Wales Hospital, Sydney, NSW 2031, Australia

**Keywords:** Endoglin, Nitric oxide, FLK1, SMAD2, Hemogenic endothelium, Hematopoiesis

## Abstract

During embryonic development, hematopoietic cells develop by a process of endothelial-to hematopoietic transition of a specialized population of endothelial cells. These hemogenic endothelium (HE) cells in turn develop from a primitive population of FLK1^+^ mesodermal cells. Endoglin (ENG) is an accessory TGF-β receptor that is enriched on the surface of endothelial and hematopoietic stem cells and is also required for the normal development of hemogenic precursors. However, the functional role of ENG during the transition of FLK1^+^ mesoderm to hematopoietic cells is ill defined. To address this we used a murine embryonic stem cell model that has been shown to mirror the temporal emergence of these cells in the embryo. We noted that FLK1^+^ mesodermal cells expressing ENG generated fewer blast colony-forming cells but had increased hemogenic potential when compared with ENG non-expressing cells. TIE2^+^/CD117^+^ HE cells expressing ENG also showed increased hemogenic potential compared with non-expressing cells. To evaluate whether high ENG expression accelerates hematopoiesis, we generated an inducible ENG expressing ES cell line and forced expression in FLK1^+^ mesodermal or TIE2^+^/CD117^+^ HE cells. High ENG expression at both stages accelerated the emergence of CD45^+^ definitive hematopoietic cells. High ENG expression was associated with increased pSMAD2/eNOS expression and NO synthesis in hemogenic precursors. Inhibition of eNOS blunted the ENG induced increase in definitive hematopoiesis. Taken together, these data show that ENG potentiates the emergence of definitive hematopoietic cells by modulating TGF-β/pSMAD2 signalling and increasing eNOS/NO synthesis.

## INTRODUCTION

Endoglin (ENG), also known as CD105, is a 190 kDa homodimeric transmembrane glycoprotein composed of disulfide-linked subunits (Gougos and Letarte, 1988). It was first identified on the surface of pre-B acute lymphoblastic cells (Zhang et al., 1996), and was later found abundantly expressed on the surface of vascular endothelial cells (Cheifetz et al., 1992). ENG functions as an auxiliary receptor that modulates TGF-β signaling and promotes endothelial cell proliferation (Barbara et al., 1999). During development, ENG is first detected at the onset of gastrulation in the extra-embryonic ectoderm and future amniotic fold at embryonic day (E) 6.5. At E7–7.5, expression extends to the amnion and allantois and is also observed in hematopoietic and endothelial cells of yolk sac blood islands (Ema et al., 2006). By E9.5, ENG is expressed throughout the developing vasculature and mesenchyme, but is absent from epithelial cells (Jonker and Arthur, 2002). In addition to its ubiquitous expression on the surface of proliferating endothelial cells, ENG is also expressed on endothelial cells and hematopoietic clusters within the dorsal aorta (DA), which contain emerging hematopoietic stem cells (HSCs) in the developing embryo (Cheifetz et al., 1992; Perlingeiro, 2007), and on the surface of adult bone marrow long-term HSCs (Chen et al., 2002).

The mesoderm that contributes to blood and endothelial cells of the yolk sac blood islands represents a rare population that migrates from the posterior primitive streak. Whether the earliest blood and endothelial cells originate from a shared mesodermal precursor, i.e. a hemangioblast, or from distinct mesodermal precursors is a moot point. Whereas *in vitro* differentiation of embryonic cell populations and *in vivo* labelling in zebrafish support the existence of a shared progenitor (Huber et al., 2004; Vogeli et al., 2006), *in vivo* labelling and cell tracing in mice support largely independent origins (Padrón-Barthe et al., 2014). However, labelling rapidly dividing heterogeneous cell populations in E5.5–7.5 mouse embryos runs the risk of reporter systems marking a mix of epiblast, mesodermal, blood and endothelial progenitors and a method to uniquely label epiblast cells and trace their progeny *in vivo* remains elusive. Nevertheless, a clonal assay that permitted isolation of murine blast colony–forming cells (BL-CFCs) has been used extensively to define the presence of and quantify hemangioblasts *in vitro* and *in vivo* (Choi et al., 1998; Huber et al., 2004; Kennedy et al., 1997). In the presence of VEGF, BL-CFCs form blast colonies which upon re-plating give rise to primitive and definitive blood progenitors and endothelial cells (Choi et al., 1998; Kennedy et al., 1997). Blast colonies express a number of genes common to both hematopoietic and endothelial lineages, including *Scl*, *CD34*, and *Flk1* (Kennedy et al., 1997).

The close spatio-temporal association between ENG expression and the emergence of hemato-endothelial tissues during development (Ema et al., 2006; Roques et al., 2012) led to investigations into a possible functional role for *Eng* in the embryonic emergence of blood and endothelium (Borges et al., 2012; Perlingeiro, 2007; Zhang et al., 2011). These investigations showed that ENG null embryonic stem (ES) cells had a decreased ability in generating BL-CFC, and demonstrated reduced primitive erythroid and angiogenic differentiation potential (Perlingeiro, 2007; Choi et al., 1998). Myelopoiesis and definitive erythropoiesis were also severely impaired in the absence of ENG but lymphopoiesis was only mildly reduced (Cho et al., 2001). The absence of ENG however did not appear to perturb expression of early mesodermal markers such as *Brachyury* and *Flk1* (Perlingeiro, 2007; Cho et al., 2001). Taken together, these data suggested that ENG plays a role during commitment of mesodermal precursors to the hematopoietic fate. However, the precise nature of this role and how ENG promotes hematopoiesis during early embryonic development are unknown.

In this study, we have taken advantage of the embryoid body (EB) and liquid culture differentiation systems using ES cells (Fehling et al., 2003; Lancrin et al., 2009) to functionally evaluate the hemogenic potential of ENG expressing and non-expressing cell fractions at different stages of embryonic blood development. We show that ENG expression in FLK1^+^ cells mark a population of cells with early hemogenic and hematopoietic potential. We also show using an ES cell line engineered to overexpress ENG under Doxycycline (Dox) control that ENG drives the acceleration of hemogenic commitment of FLK1^+^ cells and definitive hematopoiesis and that it does so by increasing nitric oxide (NO) levels via pSMAD2 signaling and increased eNOS expression.

## RESULTS

### ENG expressing cells are abundant prior to FLK1 expression but do not contribute to hematopoiesis.

ENG expression has been reported to both be associated with and also required for normal hemangioblast development (Perlingeiro, 2007; Borges et al., 2013). However, the role of ENG during different stages of hematopoietic commitment is unclear. To evaluate ENG expression during ES/EB differentiation, we used the *Bry*-GFP ES cell line, which has previously been used to characterise the developmental transition of mesodermal precursors (GFP^+^/FLK1^−^) to hemangioblasts (GFP^+^/FLK1^+^) to committed progenitors (GFP^−^/FLK1^+^) (Fehling et al., 2003). ENG is expressed in ∼90% of undifferentiated ES cells but the proportion of ENG expressing cells fall during ES/EB differentiation and plateau at ∼days 3–4 (supplementary material Fig. S1). However, as the frequency of FLK1^+^ cells increase at day 5, ENG co-expression also increased (supplementary material Fig. S1). As expected, *Bry*-GFP^+^ cells harvested from day 2 EBs progressively upregulated FLK1, whereas *Bry*-GFP^−^ cells, whether they co-expressed ENG or not, failed to do so (supplementary material Fig. S2A). This data shows that early ENG expression is not a determinant of later acquisition of BRY or FLK1 expression (supplementary material Fig. S2A). Furthermore, ENG expression does not appear to affect the inability of FLK1^−^ cells to generate CD41^+^ hematopoietic cells (supplementary material Fig. S2B).

### ENG expression on FLK1+ cells is accompanied by reduced BL-CFC potential and early commitment towards the hematopoietic lineage

**Fig. 1.**
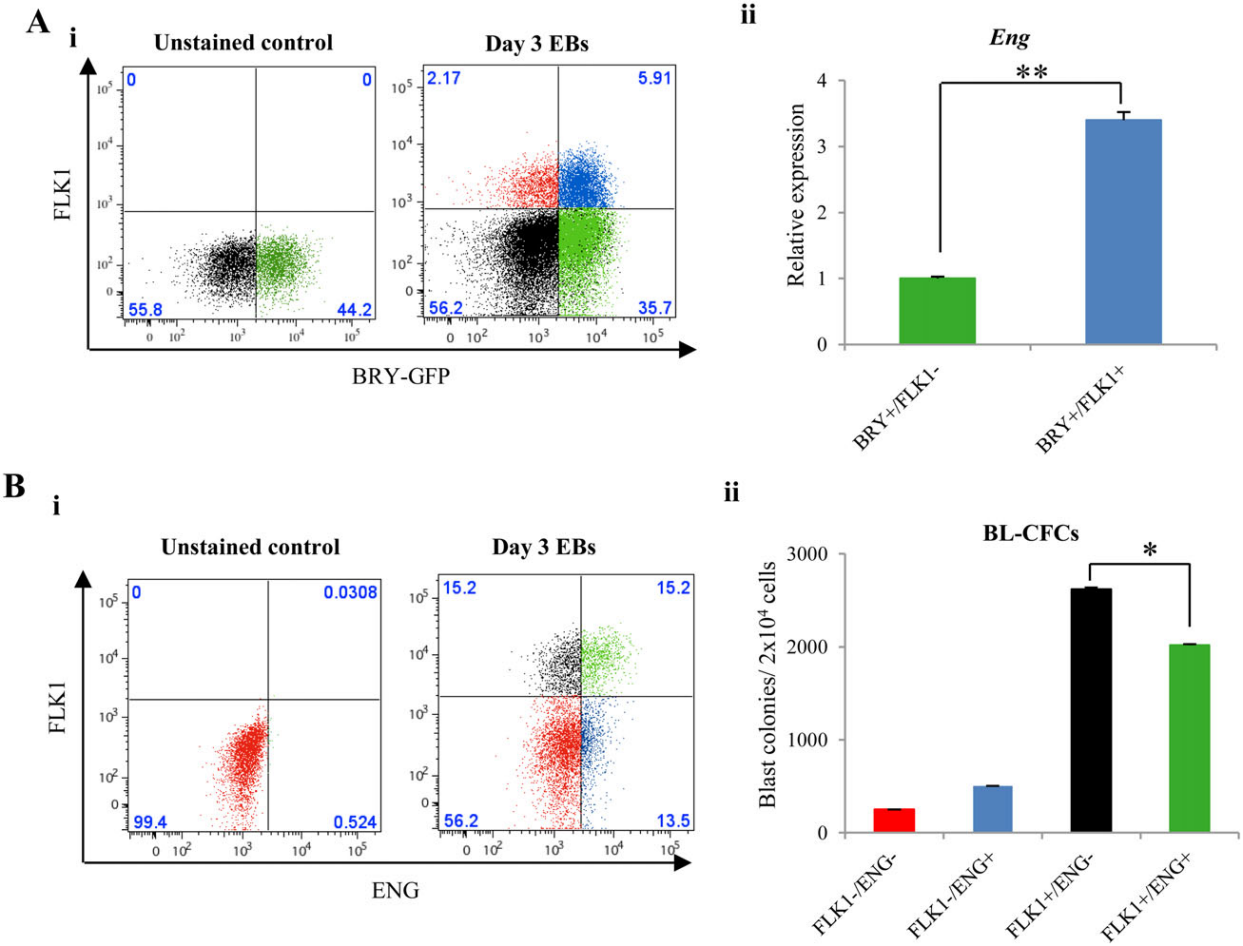
**ENG expression on FLK1^+^ mesodermal cells is associated with reduced BL-CFC potential.** (A) (i) A representative graph showing *Bry*GFP and FLK1 expression by flow cytometry in day 3 EBs. (ii) *Eng* mRNA expression in sorted BRY^+^/FLK1^−^ and BRY^+^/FLK1^+^ fractions from day 3 EBs. (B) (i) A representative flow cytometry graph showing surface expression of ENG and FLK1 in day 3 EBs. (ii) Bar chart showing the number of blast colonies generated from cell fractions sorted from each quadrant in Bi. The error bars represent SEM values across three independent biological replicates each performed in triplicate. The *P* values were calculated using a student's *t*-test. *=*P*<0.05; **=*P*<0.01.

At day 3 of EB differentiation, Bry-GFP^+^/FLK1^+^ mesodermal cells express threefold more *Eng* than their FLK1^−^ counterparts (Fig. 1Ai,ii). Furthermore, flow cytometry data show that ENG expression within the FLK1^+^ population is not uniform, where ∼50% of the FLK1^+^ cells do not express ENG (Fig. 1Bi). BL-CFC assays performed with cells sorted from each quadrant in Fig. 1Bi showed that ENG expression on FLK1^+^ cells in day 3 EBs marked a population with reduced BL-CFC potential compared with FLK1^+^ cells that lacked ENG expression (Fig. 1Bii). This observation was also apparent in cell fractions sorted from day 2.5 EBs (supplementary material Fig. S3) and was consistent across three different murine ES cell lines (data not shown).

**Fig. 2.**
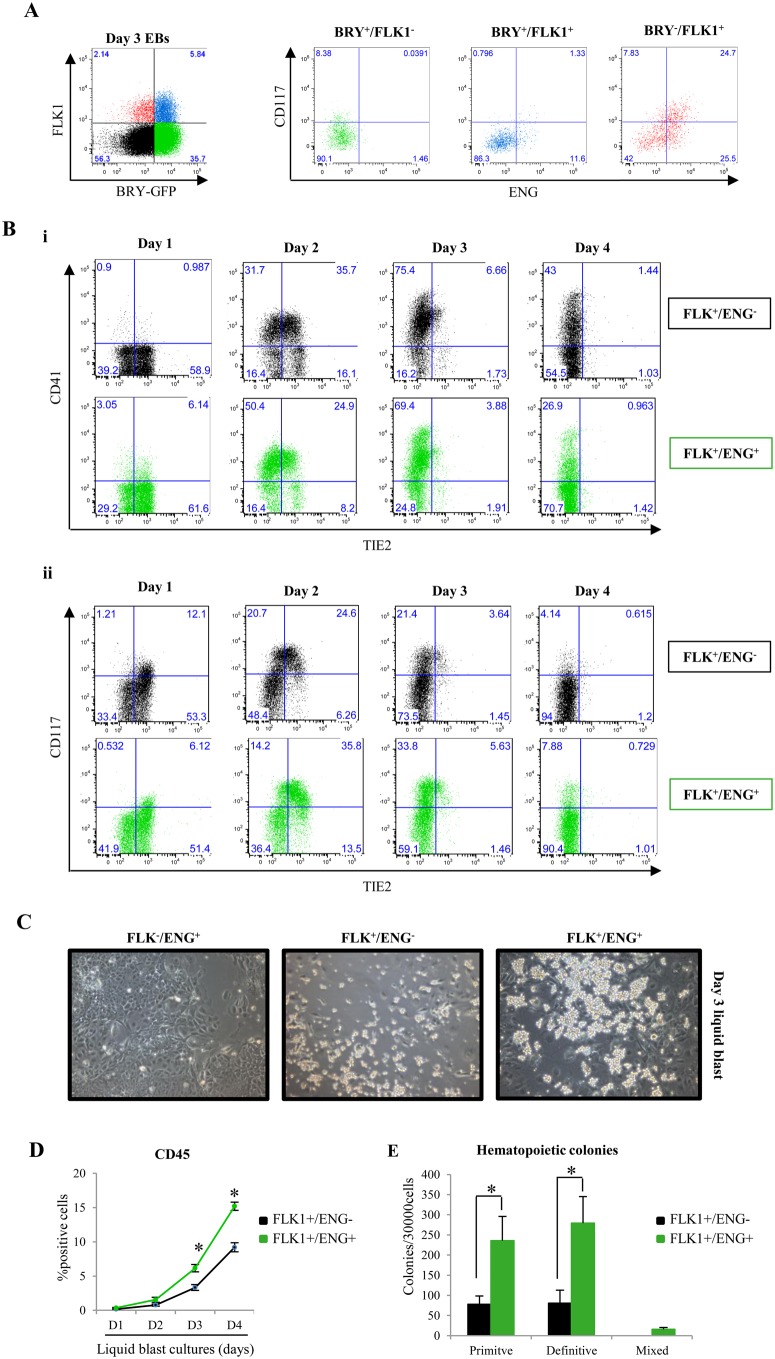
**ENG expression on FLK1^+^ mesodermal cells is associated with early commitment to a hematopoietic fate.** (A) Flow cytometry graphs showing surface expression of ENG and CD117 in day 3 BRY^+^/FLK1^−^, BRY^+^/FLK1^+^ and BRY^−^/FLK1^+^ cell populations. (B) (i) Representative flow cytometry graphs showing the expression of CD41 and TIE2 in FLK1^+^/ENG^+^ and FLK^+^/ENG^−^ sorted cell fractions during a four day liquid blast culture assay. (ii) Corresponding flow cytometry graphs showing the expression of CD117 and TIE2 in cell populations in Bi. (C) Phase-contrast photographs of FLK1^−^/ENG^+^, FLK1^+^/ENG^−^ and FLK1^+^/ENG^+^ cells at day 3 of blast culture. (D) Histogram showing the percentage of cells expressing CD45 in cell populations in Bi. The *P* values represent the statistical significance of CD45 expressing cells between two different cell populations at the same time point. (E) Bar chart showing the number of hematopoietic colonies derived from the FLK1^+^/ENG^+^ and FLK^+^/ENG^−^ cell fractions collected at day 2 of liquid blast cultures. Each experiment was performed in triplicate. The error bars represent the SEM across three independent experiments. The *P* values were calculated using student's *t*-tests from three independent biological replicates. *P<0.05.

FLK1^+^ cells have been shown to lose Bry-GFP expression and acquire C-KIT (CD117) as they commit to a hemogenic fate (Pearson et al., 2010). ENG expression preceded CD117 expression and was enhanced in day 3 Bry-GFP^−^/FLK1^+^ cells (Fig. 2A). FLK1^+^/ENG^+^ cells were also enriched with hemato-endothelial gene expression (supplementary material Fig. S4) when compared with FLK1^+^/ENG^−^ cells. To investigate whether the reduced BL-CFC potential of FLK1^+^/ENG^+^ cells was indicative of a cell population that had advanced towards a hemogenic fate, FLK1^+^/ENG^−^ and FLK1^+^/ENG^+^ cells were sorted and cultured in liquid BL-CFC media for four days to characterise the dynamics of TIE2, CD117 and CD41 acquisition (Fig. 2Bi,ii). This culture system has previously been used to show that a subset of TIE2^hi^/CD117^+^/CD41^−^ (HEI) cells represent a population of hemogenic endothelial (HE) cells, that progressively generate a TIE2^+^/CD117^+^/CD41^+^ (HEII) population followed by CD41^+^/CD45^−^ primitive and CD41^+^/CD45^+^ definitive hematopoietic cells (Lancrin et al., 2009). The developmental transition from HEI to HEII and Tie2^−^/CD41^+^ hematopoietic cells was accelerated in FLK1^+^/ENG^+^ cells (35.8% and 50.4% at day 2) when compared with FLK1^+^/ENG^−^ cells (24.6% and ∼30%) (Fig. 2Bi,ii). There was also an increase in total hematopoietic cell number as evident in phase-contrast photographs of the liquid cultures with significantly more hematopoietic cell formation in FLK1^+^/ENG^+^ than in FLK1^+^/ENG^−^ cultures at a comparable stage (Fig. 2C). There was an accompanying increase in the frequency of CD45^+^ cells at each stage in FLK1^+^/ENG^+^ liquid cultures compared with FLK1^+^/ENG^−^ cultures (Fig. 2D).

To evaluate this in more detail, cells were collected from day 2 liquid blast cultures and seeded into hematopoietic CFU assays. Significantly more primitive and definitive colonies were generated from the FLK1^+^/ENG^+^ fraction compared with the FLK1^+^/ENG^−^ fraction (Fig. 2E). There was no skewing of differentiation towards any particular lineage (supplementary material Fig. S5). No hematopoietic colonies were detected in the FLK1^−^ fractions regardless of ENG expression. It is salient to note however that FLK1^+^/ENG^−^ cells do not remain ENG^−^ for long and rapidly express ENG and at 24 hours there is little difference in ENG expression between pre-culture FLK1^+^/ENG^−^ and FLK1^+^/ENG^+^ cells in liquid culture (supplementary material Fig. S6). Taken together with the decrease in BL-CFC potential in FLK1^+^/ENG^+^ cells compared with FLK1^+^/ENG^−^ cells, these data suggest that surface expression of ENG in FLK1^+^ cells marks a population of cells that are more advanced in their development towards a hematopoietic fate.

### Induced ENG expression in FLK1^+^ mesoderm increases the frequency of hemogenic endothelial cells and enhances definitive hematopoiesis

To establish whether ENG expression merely accompanies or drives the acceleration of hematopoietic commitment of FLK1^+^ cells, we generated an inducible ENG ES cell line (i*Eng*:GFP) using parental LoxP-Cre Ainv18 ES cells (Kyba et al., 2002) in which ENG is linked to GFP via a 2A peptide to facilitate monitoring (supplementary material Fig. S7). The addition of Dox at day 0 of ES cell differentiation resulted in the induction of GFP and sustained surface expression of ENG (supplementary material Fig. S8Ai,ii). In addition to the proportion of cells expressing ENG, the abundance of *Eng* transcripts in Flk1^+^ cells was also increased (supplementary material Fig. S8Aiii). As previously reported, we also observed that *Bry* expression levels and the proportion of FLK1 expressing cells were largely unaltered with ENG overexpression (Baik et al., 2012) (supplementary material Fig. S8B). When DOX was added at day 0 to differentiating *iEng* ES cells and BL-CFC assays were performed at days 2 and 3, we saw less BL-CFCs in DOX treated cells compared with untreated cells (supplementary material Fig. S9). As ENG is expressed in undifferentiated ES cells and expression falls during ES differentiation (supplementary material Fig. S1) forced expression of ENG prior to the emergence of FLK1^+^ mesoderm probably skews differentiation towards cell fates other than those derived from progenitors that give rise to blast colonies.

**Fig. 3.**
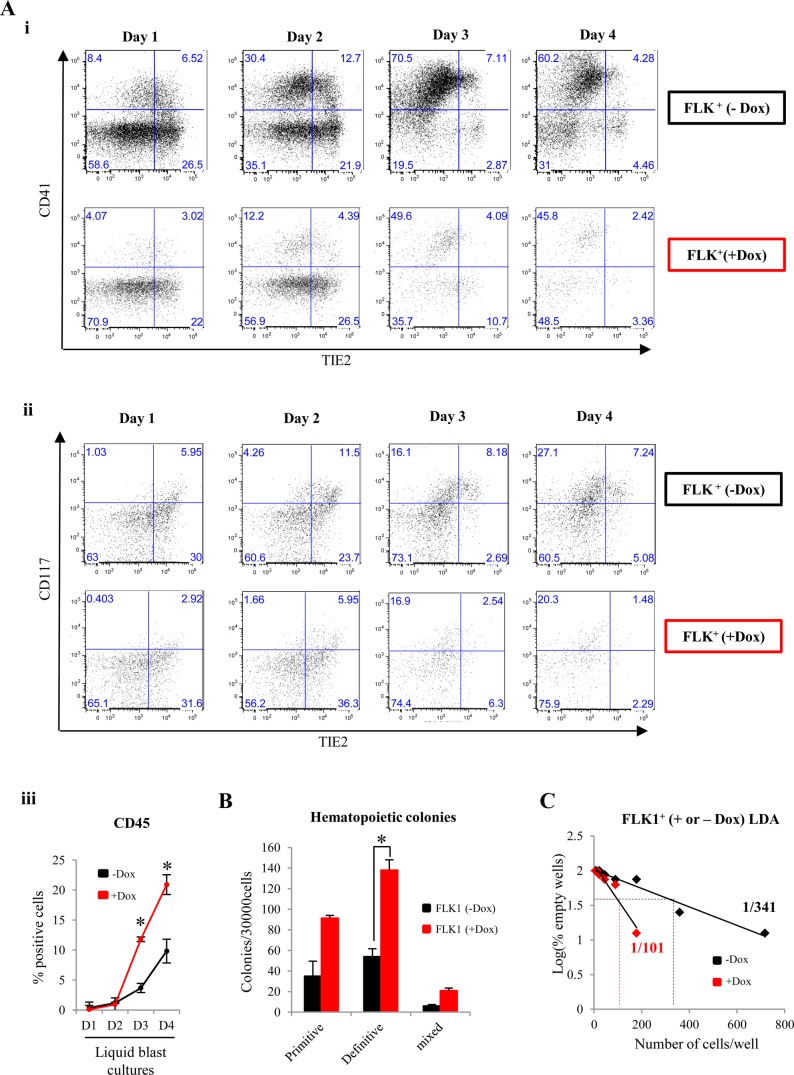
**Induction of high ENG expression in FLK1^+^ cells in day 3 EBs enhances their hemogenic potential.** i*Eng*:GFP ES cells were differentiated into day 3 EBs and FLK1 positive cells were sorted and cultured (with or without Dox) for four days in media supporting blast colony differentiation. (A) (i) Representative flow cytometry graphs showing CD41 and TIE2 surface expression during four days of liquid blast cultures of FLK1^+^ cells with Dox (red) or without Dox (black). (ii) Representative flow cytometry graphs showing CD117 and TIE2 surface expression from the same cell populations in Ai. (iii) Histograms showing the percentage of cells expressing CD45 during 4 days of liquid blast culture. The error bars represent SEM values calculated from three independent biological replicates. (B) Bar chart showing the number of hematopoietic colonies in cells isolated from day 2 of culture. (C) FLK1^+^ cells sorted from day 3 i*Eng*:GFP EBs were seeded on to a 96 well plate in a limiting dilution assay (LDA) to quantify the effect of Dox induction on the frequency of HE. The histograms represent the data obtained from the LDA which shows that the frequency of HE increases from 1:341 (black) to 1:101 (red) following induction of ENG. The P values were calculated using *t*-tests from three independent biological replicates. *P<0.05.

To investigate the effects of ENG induction in FLK1^+^ mesoderm, FLK1^+^ cells were sorted from day 3 i*Eng*:GFP EBs and cultured in the presence or absence of Dox in four day liquid cultures (Fig. 3A). High ENG expression in FLK1^+^ cells resulted in comparatively lower frequencies of TIE2^+^/CD117^+^ cells and CD41^+^ cells (Fig. 3Ai,ii) but increased frequencies of CD45^+^ cells from day two onwards (Fig. 3Aiii). Cells collected at day 4 were also seeded into hematopoietic CFU assays, and showed a significant increase in the number of definitive hematopoietic colonies following *Eng* induction (Fig. 3B). As with endogenous ENG^+^ versus ENG^−^ fractions in Fig. 2E and supplementary material Fig S5, there was no skewing of haematopoiesis towards any particular lineage (supplementary material Fig. S10). To investigate the relationship between the decrease in frequencies of TIE2^+^/CD117^+^ and CD41^+^ cells and the increase in CD45^+^ cell frequencies and definitive hematopoietic colonies, we quantified the frequency of functional HE progenitors following Dox treatment. To this end, a limiting dilution assay (LDA) was performed with sorted FLK1^+^ cells in the presence or absence of Dox. After four days, wells containing cells with hematopoietic morphology were counted. Following ENG induction in FLK1^+^ cells the frequency of functional HE cells increased from 1:341 (untreated) to 1:101 (treated) (Fig. 3C). These data show that high ENG expression in FLK1^+^ cells yields higher numbers of hemogenic progenitors within a diminished pool of TIE2^+^/CD117^+^ cells. Although ENG expression in FLK1^+^ mesoderm potentiates hematopoiesis, it is not a pre-requisite for HE commitment and haematopoiesis. FLK1^+^ mesodermal cells from day 3 EBs from ENG^−/−^ ES are still able to generate HE and blood cells in liquid cultures but their numbers, as shown by LDA and colony counts are severely compromised (supplementary material Fig. S11).

### Induced ENG expression in hemogenic endothelial progenitors enhances definitive hematopoiesis

**Fig. 4.**
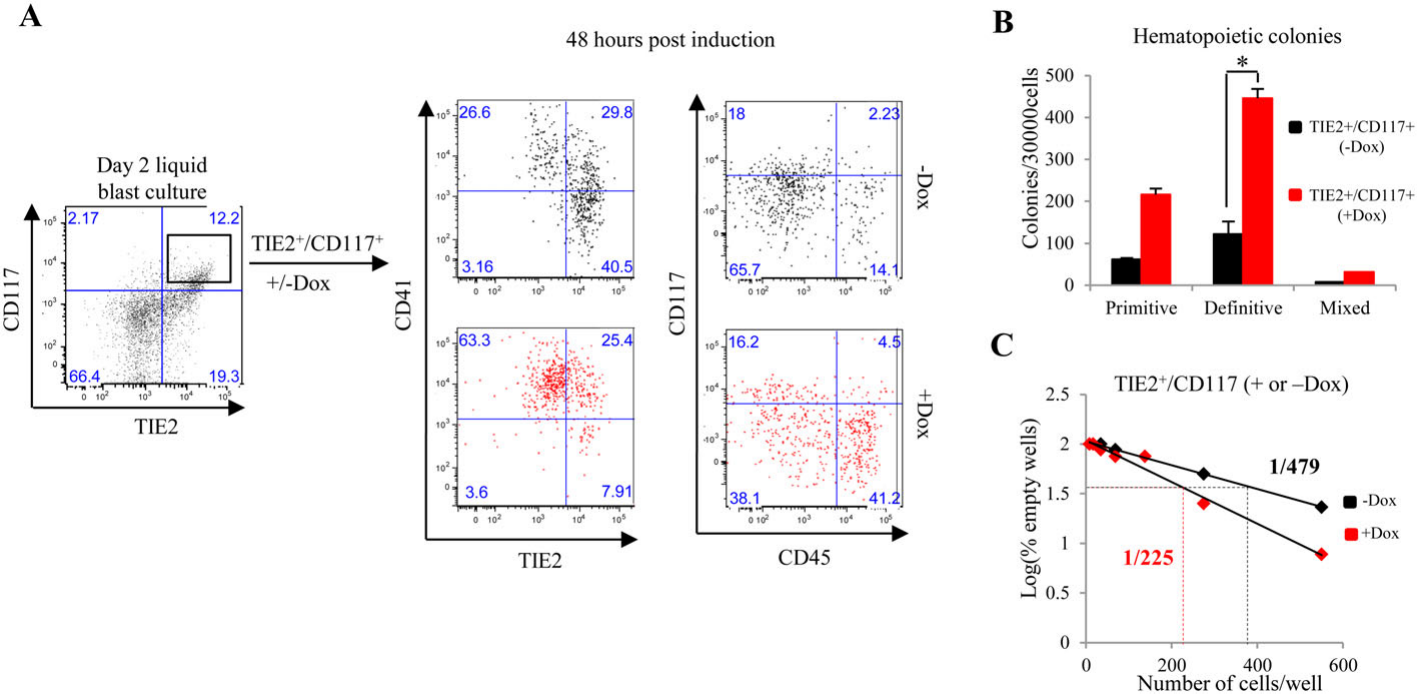
**Induction of high ENG expression in TIE2^+^/CD117^+^ cells results in the loss of endothelial markers and an increase in hematopoietic colony formation.** (A) A representative flow cytometry graph showing TIE2 and CD117 expression at day 2 of liquid blast culture of FLK1^+^ cells harvested from day 3 *iEng* EBs. TIE2^+^/CD117^+^ cells were sorted and re-plated in liquid blast media for a further 48 hours in the presence or absence of Dox. The flow cytometry graphs to the left show surface expression of CD41 and TIE2 in each fraction and the graphs to the right show surface expression of CD117 and CD45. (B) Bar chart showing the number of hematopoietic colonies generated from cells collected from A. The error bars represent SEM values calculated from three independent biological replicates performed in triplicate. (C) TIE2^+^/ CD117^+^ cells sorted from day 2 liquid blast cultures were seeded on to a 96 well plate in a limiting dilution assay (LDA) to quantify the effect of ENG induction on the hematopoietic differentiation potential of TIE2^+^/CD117^+^ HE cells. The histograms represent the data obtained from the LDA which shows that the frequency of blood emergence from HE cells increases from 1:479 (black) to 1:225 (red) upon induction of ENG. The *P* values were generated from three independent biological replicates using a student *t*-test. *P<0.05.

To determine if ENG can similarly impact on the kinetics of blood emergence if overexpressed at later time points, we sorted TIE2^+^/CD117^+^ cells from day 2 liquid blast cultures and re-plated them in the presence or absence of Dox (Fig. 4A). At 48 hours post-induction, the GFP^+^ fraction was analysed by flow cytometry. There were increased frequencies of CD41^+^ (88.3% versus 54.4%) and CD45^+^ (45.2% versus 16.3%) cells and reduced frequencies of TIE2^+^ (28.7% and 66.2%) cells following ENG induction (Fig. 4A). TIE2^+^/CD117^+^ cells cultured in the presence or absence of Dox for two days were also seeded into hematopoietic CFU assays. Again, there was significant increase in the numbers of definitive hematopoietic colonies following ENG induction (Fig. 4B). To further evaluate whether ENG overexpression in TIE2^+^/CD117^+^ cells enriched numbers of functional hemogenic progenitors, we performed LDAs with sorted TIE2^+^/CD117^+^ cells with or without Dox. At 48 hours, wells containing cells with hematopoietic morphology were quantified. The number of blood producing hemogenic progenitors had increased from 1:479 (untreated) to 1:225 (treated) (Fig. 4C). Taken together, these data show that induction of ENG expression in FLK1^+^ cells or TIE2^+^/CD117^+^ promoted hematopoietic commitment and the emergence of CD45^+^ hematopoietic cells.

### ENG induced acceleration of definitive hematopoiesis is mediated by increased eNOS/NO activity

**Fig. 5.**
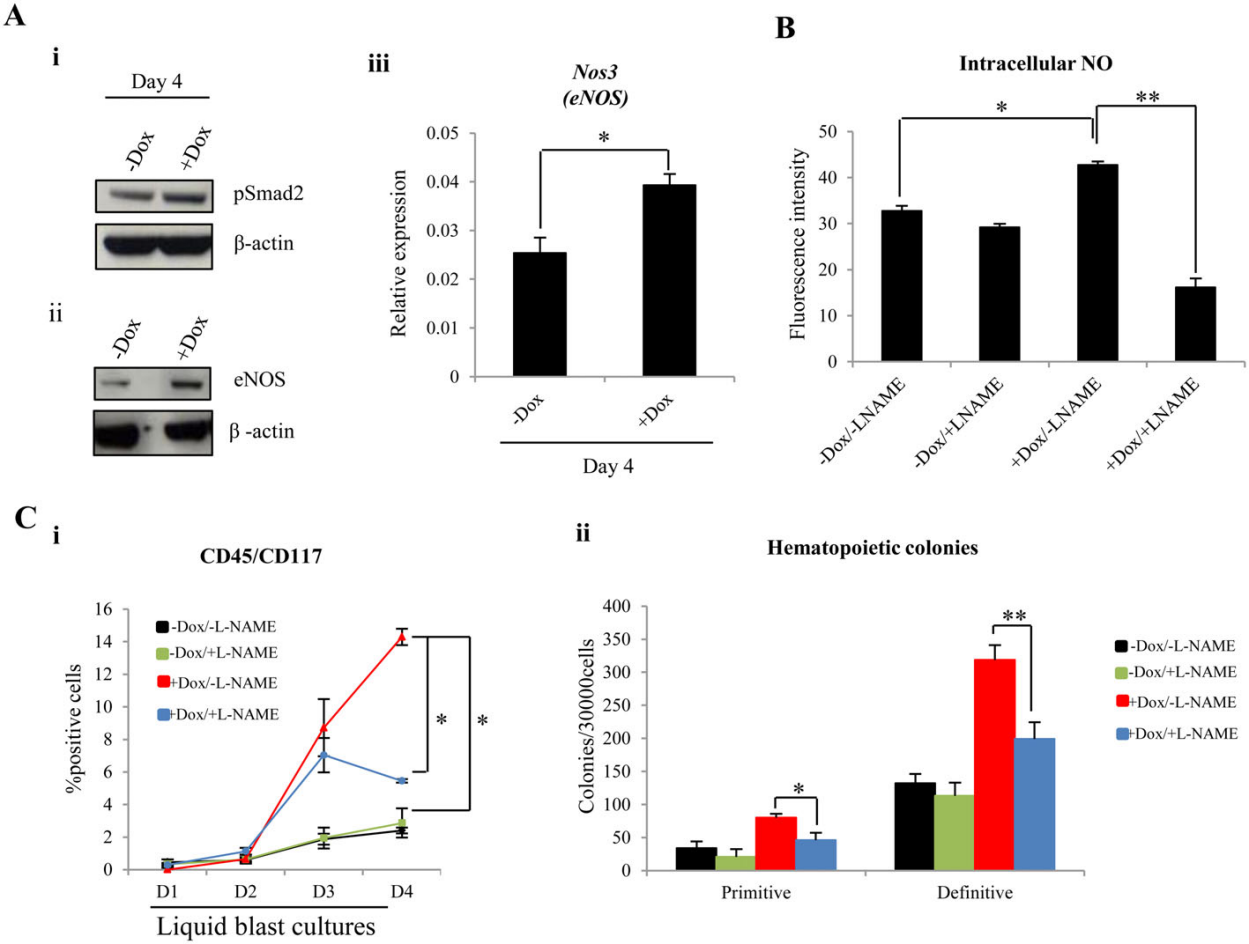
**ENG induced acceleration of definitive hematopoiesis is mediated by increased eNOS/NO activity.** (A) Western blots showing Smad2 (i) and eNOS (ii) expression relative to β-actin in cells isolated at day 4 from liquid blast cultures of Dox treated and untreated iEng FLK1^+^ cells. (iii) RT-PCR showing Nos3 (eNOS) mRNA levels in FLK1^+^ cells after four days of treatment with or without Dox. (B) Bar chart showing the levels NO in cells harvested at day 3 from liquid cultures of iEng:GFP FLK1^+^ cells with or without Dox treatment and in the presence or absence of L-NAME (2 mM final), an inhibitor of NOS enzymes. (C) (i) Histogram showing ENG induced increase in CD45^+^/CD117^+^ hematopoietic cell emergence in liquid blast cultures and its modulation by L-NAME (ii) Bar chart showing the number of hematopoietic colonies generated from cells harvested at day 4. The error bars represent SEM values calculated from three independent biological replicates performed in triplicate. The *P* values were generated from three independent biological replicates and were calculated using student *t*-tests. *P<0.05; **P<0.01.

There is also an extensive body of evidence showing that ENG increases eNOS expression by modulating Smad2 protein levels in endothelial cells (Santibanez et al., 2007). Shear stress induced expression of eNOS and NO production has been shown to enhance hematopoiesis in ES/EB differentiation assays and also *in vivo* (Adamo et al., 2009; North et al., 2009). Shear stress has also been shown to increase ENG expression in endothelial cells, which in turn mediates TGF-β/pSmad2 signaling and leads to the increase of eNOS (Lebrin et al., 2004; Seghers et al., 2012). Therefore, we speculated that the increase in definitive hematopoiesis that we observed with ENG induction in FLK1^+^ and TIE2^+^/CD117^+^ cells could be mediated by the ENG/pSmad2/eNOS/NO endothelial axis. Indeed, cellular levels of pSMAD2 (Fig. 5Ai) and expression levels of eNOS (*Nos3*) mRNA and protein (Fig. 5Aii,iii) were increased in ENG induced cells. Furthermore, there was no change in protein levels of SMAD2 (data not shown) and pSMAD1/5 (data not shown). Induction of ENG in FLK1^+^ cells was accompanied by an increase in intra-cellular NO levels, and this increase was blunted by the addition of NG-nitro-L-arginine methyl ester (L-NAME), a selective inhibitor of NOS enzymes (Fig. 5B). The addition of L-NAME to liquid cultures of FLK1^+^ cells also restrained the increase in ENG induced emergence of CD45^+^ cells and numbers of definitive hematopoietic colonies (Fig. 5Ci,ii).

## DISCUSSION

We have used an ES cell differentiation system to evaluate the impact of ENG expression on hematopoietic commitment in mesodermal progenitors. We report that FLK1^+^ mesodermal cells that express ENG have reduced BL-CFC potential and are committed towards a hematopoietic fate. FLK1^+^ cells from day 3 EBs subjected to high ENG expression showed increased capacity to produce hemogenic endothelial, CD45^+^ hematopoietic cells and definitive hematopoietic colonies. Forced ENG expression in TIE2^+^/CD117^+^ cells from day 2 liquid cultures also showed similar properties. ENG mediates the acceleration of hematopoiesis from mesodermal and hemogenic progenitors by facilitating increased eNOS and NO levels.

The TGF-β superfamily of ligands signal by binding a complex of type 1 and type 2 receptors and mediating phosphorylation of down-stream SMAD transcription factors to impact on gene transcription. The identity of the type 1 receptors (ALK1 or ALK5) that engage the ligand and its cognate type 2 receptor helps determine whether downstream TGF-β signaling occurs via the pSMAD1/5/8 pathway (ALK1) that promotes cell migration and proliferation or the pSMAD2/3 pathway (ALK5), which is associated with cell differentiation (Seghers et al., 2012). Shear stress induced increase in ENG expression facilitates ALK5 mediated pSMAD2 phosphorylation and eNOS expression (Santibanez et al., 2007; Seghers et al., 2012). Shear stress has also been reported to increase hematopoietic colony-forming potential and expression of hematopoietic markers in the para-aortic splanchnopleura/aorta-gonads-mesonephros of mouse embryos and that abrogation of nitric oxide, a mediator of shear-stress-induced signalling, compromises hematopoietic potential *in vitro* and *in vivo* (Adamo et al., 2009; North et al., 2009). Here we show that stage specific overexpression of ENG was associated with increased pSMAD2/eNOS and NO levels and abrogation of eNOS activity blunted the increase in definitive hematopoiesis that was mediated by high ENG. eNOS molecules have been reported to bind the cytoplasmic region of the ENG receptor, where Hsp90 molecules reside. This ENG mediated proximity of eNOS and Hsp90 generates a complex that catalyzes the degradation of L-Arginine (L-Arg) to L-Citrulline (L-Cit) and generates NO molecules. This eNOS/Hsp90 association is impaired in ENG^−/−^ ES cells, leading to decreased production of NO (Santibanez et al., 2007; Toporsian et al., 2005).

The most direct evidence to show that the diminished BL-CFC potential in a FLK1^+^ mesodermal cell that expressed ENG was accompanied by commitment towards a haemogenic fate would be to sort the same cell into both blast colony (BL-CFCs) and liquid culture (haemogenic endothelial and blood) assays. As this is beyond current limits of technology, we had to settle for sorting FLK1^+^/ENG^−^ or FLK1^+^/ENG^+^ cells from the same EB differentiation assay into either BL-CFCs (Fig. 1B) or liquid cultures (Fig. 2B). The data showed that FLK1^+^/ENG^+^ cells produced fewer blast colonies but were more advanced in expression of haematopoietic markers (CD41/45; Fig. 2B,D), emergence of blood cells (Fig. 2C,E) than FLK1^+^/ENG^−^ cells. The decrease in BL-CFCs that we observed in ENG expressing FLK1^+^ cells in day 3 EBs, contrasts with earlier reports that ENG marked a population of cells with increased BL-CFC activity (Perlingeiro, 2007). The number of BL-CFCs that can be generated from ES/EB cultures is closely linked to the emergence of FLK1^+^ cells. ES/EBs acquire FLK1 expression by day 2.5 (∼6% of cells), and this progressively increases to reach 20–30% by day 4, after which it begins to fall (Fehling et al., 2003). The number of blast colonies on the other hand peaks at day 3 and falls sharply at day 4. There is therefore a window during ES/EB differentiation, where FLK1^+^ cell numbers increase but BL-CFC potential decreases. The numbers of BL-CFCs in the report associating ENG expression with increased BL-CFC activity (Perlingeiro, 2007) (10–30 BL-CFCs/5×10^4^ FLK1^+^ cells from day 3 EBs) were considerably lower than those in this study (e.g. 2000–2500 BL-CFCs/2×10^4^ FLK1^+^ cells from day 3 EBs; Fig. 1Bii). The low numbers of BL-CFCs also coincided with the presence of an unusually high number of FLK1^+^ cells at day 3 (∼75% versus ∼25%). Taken together, these data suggest that the culture conditions might not have been ideal to assess functional differences between cell fractions.

CD117 expression on day 3 FLK1^+^ cells has been reported as an early marker of hematopoietic commitment (Kataoka et al., 1997). It is noteworthy that CD117 expression in day 3 FLK1^+^ cells is limited to a fraction of FLK1^+^/ENG^+^ cells and is not expressed on FLK1^+^/ENG^−^ cells (Fig. 2A). This again is consistent with ENG as an early marker of hematopoietic commitment within a pool of FLK1^+^ cells in day 3 EBs. ENG overexpression in bulk populations of day 4–6 EBs has been reported to have no effect on definitive hematopoietic colony formation (Baik et al., 2012). This again contrasts with the observation that ENG overexpression in relevant cell populations, i.e. FLK1^+^ cells from day 3 EBs that are enriched in hemato-endothelial progenitors or in TIE2^+^/CD117^+^ cells in day 2 liquid cultures that are enriched in hemogenic endothelial precursors, there is both an increase in definitive hematopoietic colony formation and CD45^+^ cell emergence. Only a proportion of Tie2^+^/CD117^+^ intermediates in liquid cultures possess functional HE potential (Lancrin et al., 2009). ENG overexpression in TIE2^+^/CD117^+^ cells in day 2 liquid cultures led to a decrease in CD117^+^ and 41^+^ expressing Tie2^+^ cells albeit with increased functional HE cells. Whether this is due to perturbation of surface marker expression or selection of cells that progress in culture is not known. Bearing in mind that FLK1^+^ mesoderm is closer to the apex of embryonic haematopoiesis than TIE2^+^/CD117^+^ cells, it is also interesting to note that forced expression of ENG in the former have a greater impact (∼3 fold) probably by increasing numbers of HE precursors than perturbation in the latter (∼2 fold), which have a more restricted differentiation potential. Taken together, this study underscores the importance of using refined rather than bulk cell populations when using ES/EB differentiation cultures to interpret the role of a gene during early blood development.

Overall, our data supports a role for ENG in priming the hemogenic program at a very early stage of embryonic development i.e. as early as in FLK1^+^ mesodermal progenitors. The observation that this is at least in part mediated by increased NO synthesis is particularly relevant given the role of NO in mediating shear induced HSC emergence from the aorta-gonad-mesonephros during embryonic development (Adamo et al., 2009; North et al., 2009). ENG expression in the AGM is regulated by the *ENG* promoter in conjunction with an up-stream −8 kb and two intronic +7 kb and +9 kb enhancers that collectively assemble components of the *Ets/Gata* hemogenic code (Pimanda et al., 2006; Pimanda et al., 2008). These *cis*-regulatory elements of *ENG* are bound by FLI1, GATA2 and SCL in E11.5 AGM which in turn constitute a recursively wired triad of hematopoietic transcription factors that maintain each other's expression at this site by binding their own enhancer elements in the AGM (Pimanda et al., 2007b). Constituents of the FLI1/GATA2/SCL network that regulates ENG also regulates BMP signaling and RUNX1 activity in the AGM (Narula et al., 2010; Narula et al., 2013; Pimanda et al., 2007a). RUNX1 is required for the *in vivo* generation of definitive hematopoietic cells (Okuda et al., 1996) and its expression is also shear induced (Adamo et al., 2009). Taken together with its role in potentiating NO synthesis in endothelial cells, the transcriptional connectivity of ENG expression in the AGM to a highly integrated regulatory network that governs blood emergence (Marks-Bluth and Pimanda, 2012; Pimanda and Göttgens, 2010), raises the prospect for a role for ENG in promoting the emergence of definitive hematopoietic cells from hemogenic precursors in the AGM.

## MATERIALS AND METHODS

### Murine ES cell culture and differentiation

The Bry/GFP (Fehling et al., 2003) and ENG ^−/−^ and ENG ^+/−^ (Cho et al., 2001) murine ES cells have been previously described. Murine ES cells were seeded on feeder coated plates in media composed of 84% Dulbecco's Modified Eagle Medium (DMEM, PAA Laboratories), 15% Fetal Bovine serum (FBS, Invitrogen) pre-tested for maintenance of ES cells, 0.1% of 0.15 M dilution of monothioglycerol (MTG, Sigma Aldrich) and 1000 units/ml mouse recombinant Leukaemia inhibitory factor (Esgro, Millipore). To generate EBs, cells were cultured in media consisting of IMDM supplemented with 15% FBS (PAA laboratories) pretested for efficient embryonic stem cell differentiation, 1% L-Glutamine (Invitrogen), 1% penicillin-streptomycin, 0.3% of 0.15 M MTG, 0.6% of 30 mg/ml transferrin (Life Technologies) and 1% of 5 mg/ml ascorbic acid (Sigma Aldrich). Cells were then seeded on ultra-low attachment 60 mm plates (Sterilin) and placed in a humidified incubator at 37°C and 5% CO_2_. These culture conditions were optimal for growth of day 1–5 EBs. Cells isolated from EB cultures were re-aggregated in EB media at a density of 5×10^4^ cells/ml (Sroczynska et al., 2009).

### Generating *iEng* ES cell line

*Eng* cDNA (Open Biosystems, clone MMM1013-202764815) was amplified from the pCMV-SPORT6 plasmid using 5′-GAGAGATATC-TCTCCGCCATGGACCGTGGC-3′ and 5′-GAGACTCGAGCGCCATG-CTGCTGGTGGAGC-3′ (EcoRV and XhoI restriction sites respectively) primers. The fragment was digested with EcoRV/XhoI restriction enzymes and ligated into a 2A-eGFP-pLox backbone (Kyba et al., 2002). Plasmids were sequenced using a BigDye Terminator Sequencing Kit (Life Technologies). The expression plasmid was electroporated with Cre-recombinase plasmid into Ainv18 ES cells (0.4 cm, Life Technologies) at 250 V and 450 μFaraday (201 Ω). Two days post electroporation the cells were cultured in the presence of Neomycin (480 μg/ml final; Sigma Aldrich), until the clones became visible. Primers used for sequencing were; 2A-eGFP F: ctgaaacagactttgaatttt, R: ttacttgtacagctcgtccatgcc; PA-SV40 R: tggtttgtccaaactcatcaa, pGKneo F: ctagatctcgaaggatctggag.

### Liquid BL-CFC assay

Day 3 EBs were dissociated into single cells using trypsin (Gibco), and stained with a Flk1-PE or Flk1-bio antibody (eBioscience). Sorted FLK1^+^ cells were seeded on gelatin coated plates at a density of 7.5–8.5×10^4^ cells/9.6 cm^2^ (in a humidified incubator 37°C, 5% CO_2_). The culture medium consisted of IMDM supplemented with 10% FBS pre-tested for differentiation, 1% L-Glutamine, 0.6% 30 mg/ml transferrin, 0.3% 0.15 M MTG, 0.5% 5 mg/ml ascorbic acid, 15% D4T conditioned medium, 0.1% 5 µg/ml VEGF, and 0.1% 10 µg/ml IL-6 (R&D systems).

### Methylcellulose blast colony forming (BL-CFC) assay

2×10^4^ FLK1^+^ cells collected from day 3.5–3 EBs were seeded in methylcellulose mix which consisted of IMDM supplemented with 55% methylcellulose (BioScientific), 10% FBS pretested for efficient EB differentiation, 1% L-glutamine, 1% L-glutamine, 0.6% 30 mg/ml transferrin, 0.3% 0.15 M MTG, 0.5% 5 mg/ml ascorbic acid, 15% D4T conditioned media, 0.1% 5 µg/ml VEGF, and 0.1% 10 µg/ml IL-6. A 1 ml syringe and 18 gauge blunt-end needle were used to aspirate the methylcellulose mix onto 35 mm petri dishes. The blast potential in Flk1^+^ cells was assayed in triplicate dishes of 1 ml. The plates were then placed in a humidified incubator 37°C and 5% CO_2_. Colonies were scored following four days of culture (Sroczynska et al., 2009).

### Hematopoietic methylcellulose colony-forming assay

Cells isolated from day 2 or 4 of liquid blast cultures were seeded in media containing IMDM supplemented with 55% methylcellulose, 15% plasma derived serum (PDS, Animal Technologies), 10% protein-free hybridoma medium (PFHMII, Invitrogen), 1% L-Glutamine, 0.6% 30 mg/ml transferrin, 0.3% 0.15 M MTG, 0.5% 5 mg/ml ascorbic acid, 1% 10 µg/ml kit ligand (KL), 0.1% 1 µg/ml IL-3, 0.3% 10 µg/ml G-CSF, 0.1% 5 µg/ml IL-11, 0.2% erythropoietin at 2000 units/ml, 0.2% 5 µg/ml IL-6, 0.1% 5 µg/ml TPO, and 0.1% 10 µg/ml GM-CSF. Cells were seeded at a concentration of 3×10^4^ cells/ml, and the assay was set up in triplicate dishes of 1 ml. Primitive colonies were scored after 4–5 days of culture, whereas most definitive colonies were scored after 7–10 days. All cytokines used in this assay were purchased from R&D systems (Sroczynska et al., 2009).

### Flow cytometry and cell sorting

Cells were stained on ice for 30 minutes with either a biotinylated or flurochrome-conjugated primary antibody. Dead cells were excluded from the analysis on the basis of Hoechst 33258 uptake (1 µg/ml final concentration; Invitrogen). A list of all primary and secondary antibodies can be found in supplementary material Table S1. Flow cytometry analysis was performed using standard flow cytometers; LSR or BD Canto II. Sorting was performed on an Aria II, Influx or Jazz.

### Western blot

Protein extractions were performed using RIPA lysis buffer. Protein concentrations were quantified using Bradford assay (BioRad). For Western blotting, 20–40 mg of protein per sample were loaded together with LSD Sample Buffer (Invitrogen) and antioxidant (Invitrogen) onto a 10% polyacrylamide gel (Invitrogen) and electrophoresed for 20 minutes at 80 V, followed by 60 minutes at 180 V in MOPS SDS Running buffer (Novex). The SeeBluePlus2 (Invitrogen) marker was used as a molecular weight indicator. Subsequently, proteins were transferred to nitrocellulose membrane using the iBlot dry transfer system (Invitrogen). Transferred proteins were visualized by reversibly staining membranes with Ponceau S solution (Sigma) for 15 minutes. Membrane were washed with PBS supplemented with 0.1% Tween20 (PBS/T), blocked in buffer containing PBS/T and 5% fat-free powdered milk (Marvel) or 5% BSA and blotted over-night with primary antibodies in blocking buffer. The eNOS (BD Transduction Laboratories) was used at a 1:5000 dilution. The pSmad2 and pSmad1/5 (Cell Signaling Technology) were used at 1:1000 dilution. The anti-rabbit-HRP and anti-mouse-HRP (Cell Signaling Technology) were used at 1:10000 dilution. Following three washing steps, each for 5 minutes at room temperature in PBS/T, secondary antibodies were applied. After a final set of washes, the membranes were developed with ECL Plus Western Blotting detection reagents (Amersham), according to manufacturer's protocol and the membranes were transferred to an X-ray film cassette. Films were exposed and developed in a MAS automated developing machine. Antibodies were removed from the membrane by 15 minutes incubation in stripping buffer (200 mM glycine, 0.1% SDS, 1% Tween-20, pH 2.2). After three washing steps, the membranes were blocked, as described previously, and primary antibody anti β-actin (Cell Signaling Technology; 1:10000), was applied followed by the secondary antibody.

The following antibodies were used; eNOS (BD Transduction Laboratories; cat#610296; 1:5000), pSmad2 (Cell Signaling Technology; cat#3101S; 1:1000), pSmad1/5 (Cell Signaling Technology; cat#9516S; 1:1000), Smad2/3 (Cell Signaling Technology; cat#3102S; 1:1000), β-actin (Sigma; cat#A1978; 1:10000), anti-rabbit-HRP (Life Technologies; cat#A24537; 1:10000) and anti-mouse-HRP (Cell signaling technology; cat#7076; 1:10000).

### RT-PCR

Prior to RNA isolation from samples, all equipment was treated with RNaseZap (Ambion) to remove any contaminating RNases. RNA was extracted using and RNeasy extraction kit (Qiagen) following manufacturer's instructions. Typically RNA was eluted with 30 μl RNase-free water. cDNA was then synthesised using the GoScript Reverse Transcriptase (Promega) following manufacturer's guidelines. Briefly, up to 5 μg of RNA was incubated with random primers and nuclease-free water at 70°C for 5 minutes, and then placed immediately on ice. A reverse transcription mix (5× GoScript reaction buffer, MgCl_2_, 10 mM dNTP mix, recombinant RNasin, GoScript reverse transcriptase and water) was added to the denatured RNA for amplification. The cDNA was analysed for expression using the Express SYBR Green QPCR Supermix Universal (Life Technologies), following manufacturer's guidelines.

The primers used for qPCR were; *Eng* F: aaatcccgttgcacttgg, R: actcttggctgtccttggaa; *Gata2* F: cacaagatgaatggacagaacc, R: acaggtgcccgc-tcttct; *Fli1* F: ccccagccagatccttatc, R: cagctggatctgcccact; *Nos3* F: atcca-gtgccctgcttca, R: gcagggcaagttaggatcag; *B-actin* F: tgacaggatgcagaagaaga, R: cgctcaggaggagcaatg.

### NO measurement

NO levels were measured using the OxiSelect Intracellular Nitric Oxide Assay Kit (Cell Biolabs, Inc.) according to the manufacturer's instructions. Briefly, on day 3 of liquid blast cultures, cells were harvested and replated in the presence or absence of Dox and L-NAME for 24 hours. Cells were then harvested and washed with PBS, followed by incubation with 1× NO Fluorometric probe for 2 hours at 37°C and 5% CO_2_. 1× lysis buffer was added to the cultures and incubated for 5 minutes. 150 μl of the sample suspension was transferred to black polystyrene half-area 96-well plates (Costar) and fluorescence was measured at 485 nm/520 nm. The levels of NO were calculated relative to fluorescence from the corresponding untreated cells (without probe).

### Statistical analysis

For each experiment statistical analysis was performed by calculating the standard error of the mean (SEM) across three independent experiments each performed in triplicate for colony assays, and three independent experiments for analyses of change in cell frequency over time between different treatment conditions. Statistical significance of the results was determined using unpaired *t*-tests. All *P* values were two-tailed and <0.05 and <0.001 were considered significant (*) and highly significant (**) respectively.

## Supplementary Material

Supplementary Material
