# Supplementary material for: Endoglin potentiates nitric oxide synthesis to enhance definitive hematopoiesis
Source: Biol Open. 2015 May 15;4(7):819–29. doi: 10.1242/bio.011494 (PMC4571086; doi:10.1242/bio.011494)
Supplement: Supplementary Material [file supp_4_7_819__index.html]

Endoglin potentiates nitric oxide synthesis to enhance definitive hematopoiesis — Endoglin potentiates nitric oxide synthesis to enhance definitive hematopoiesis — Supplementary Material 

# Endoglin potentiates nitric oxide synthesis to enhance definitive hematopoiesis

## BIO011494 Supplementary Material

- Supplementary Material
