# Supplementary material for: Endoglin potentiates nitric oxide synthesis to enhance definitive hematopoiesis
Source: Biol Open. 2015 May 15;4(7):819–29. doi: 10.1242/bio.011494 (PMC4571086; doi:10.1242/bio.011494)
Supplement: Supplementary Material [file supp_bio.011494_BIO011494supp.pdf]

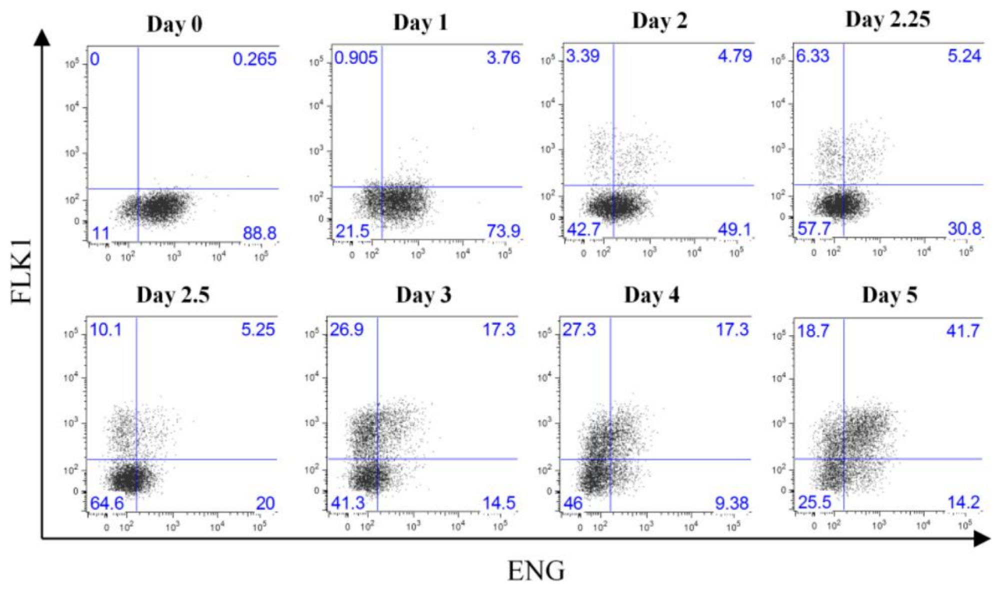


**Fig. S1. Representative flow cytometry of FLK1 and ENG expression on single cell suspensions obtained from day 1-day 5 EBs.**

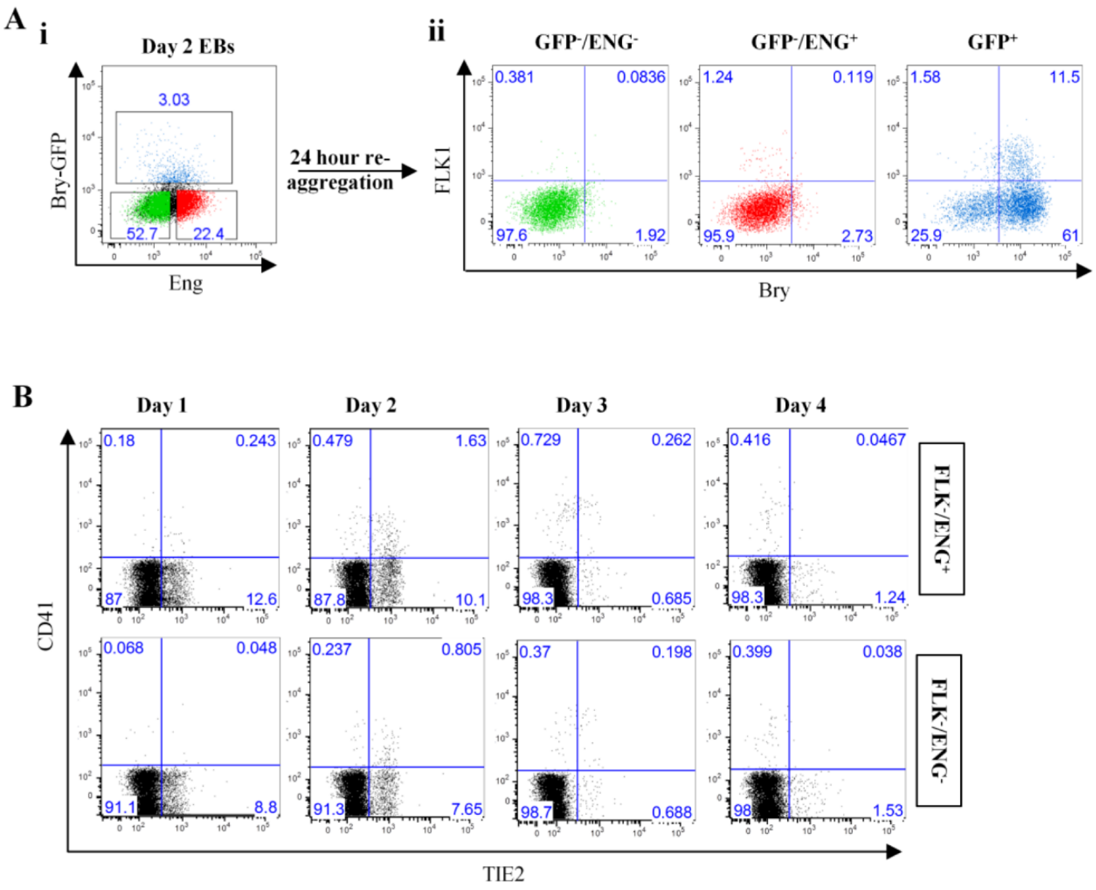


**Fig. S2.** (A) Representative flow cytometry plot showing the expression of ENG and Bry-GFP in day 2 EBs (left panel). $5\times10^4$ GFP$^-$/ENG$^-$ (Green), GFP$^-$/ENG$^+$ (Red) and GFP$^+$ (Blue) sorted cells were seeded in re-aggregation media for 24 hours. Representative flow cytometry graphs showing the expression of Bry-GFP/FLK1 for each sorted cell fraction (right panels). (B) Representative flow cytometry showing expression of TIE2 and CD41 in FLK1$^-$/ENG$^+$ and FLK1$^-$/ENG$^-$ cells collected from day 3 EBs.

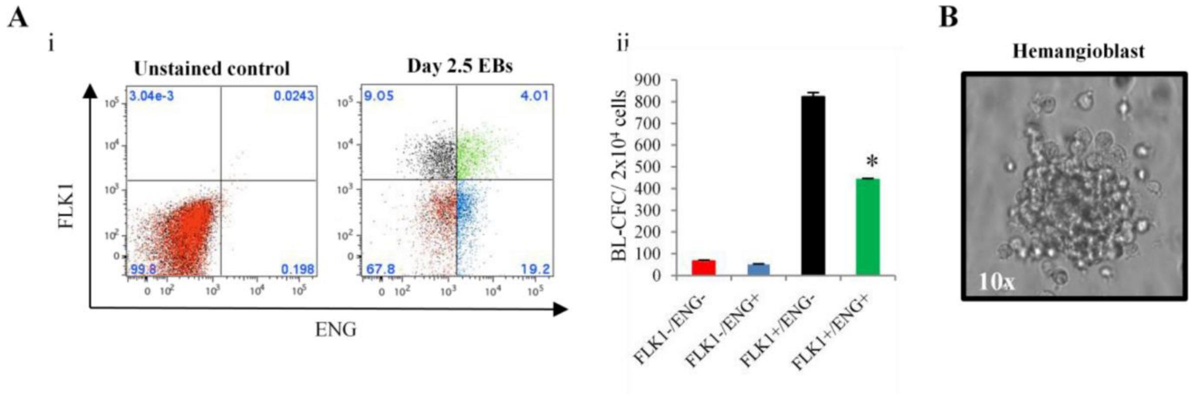


**Fig. S3.** (A) (i) Flow cytometry graph showing the expression of ENG and FLK1 in day 2.5 EBs. (ii) Bar chart showing the number of BL-CFCs in cell fractions sorted from day 2.5 EBs. 20,000 cells were plated in BL-CFC methylcellulose media, and colonies were counted after four days of culture. (B) An image showing the typical morphology of a BL-CFC at 10×magnification. *$P$<0.05.

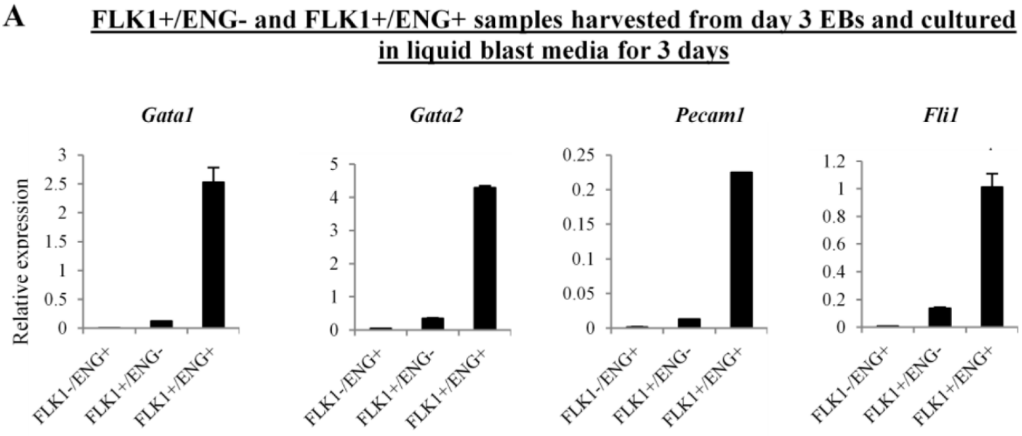


**Fig. S4.** (A) RT-PCR performed on RNA isolated from $FLK1^+/ENG^-$ and $FLK1^+/ENG^+$ samples collected at day 3 of liquid blast cultures. mRNA expression was tested for genes involved in hemangioblast specification, Gata1, Gata2, Pecam1 and Fli1. Gene expression was quantified relative to β-actin.

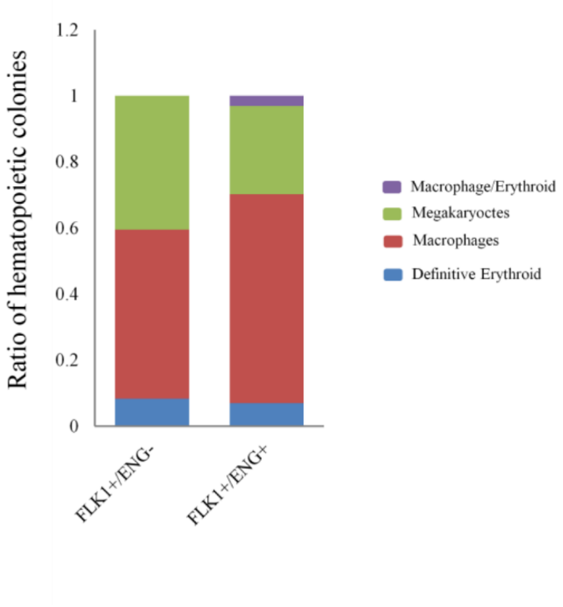


**Fig. S5. Ratios of definitive hematopoietic colonies generated by the FLK1⁺/ENG⁻ and FLK1⁺/ENG⁺ cells.**

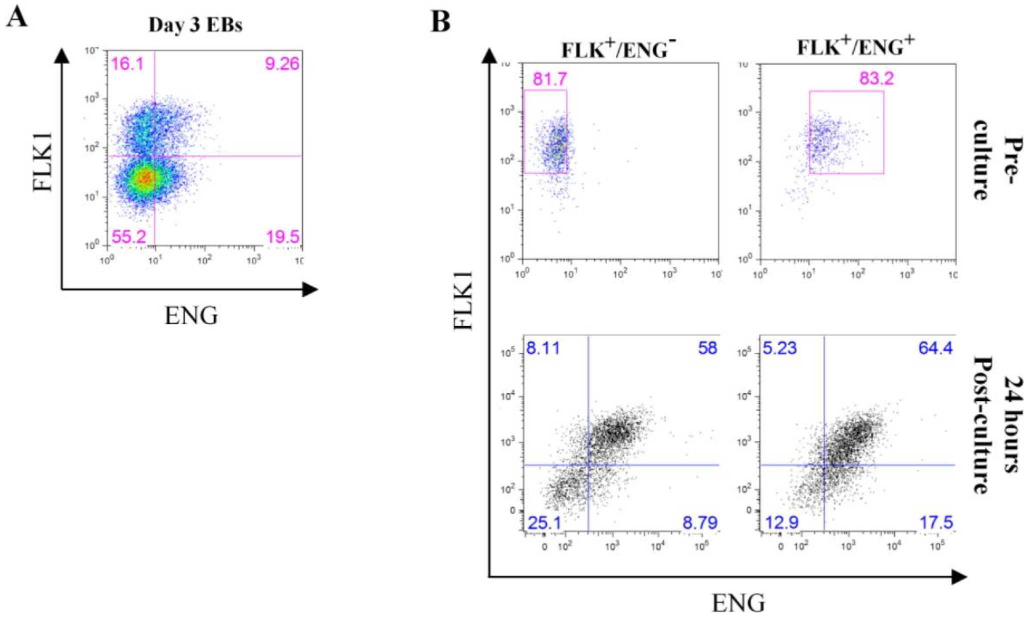


**Fig. S6. FLK1⁺/ENG⁻ cells upregulate ENG expression in liquid culture.** (A) Flow plot showing the expression of FLK1 and ENG in day 3 EBs. (B) Flow plots showing the expression of FLK1 and ENG in FLK1⁺/ENG⁻ and FLK1⁺/ENG⁺ sorted cells pre-culture (Upper panel) and 24 hours post-culture (lower panel).

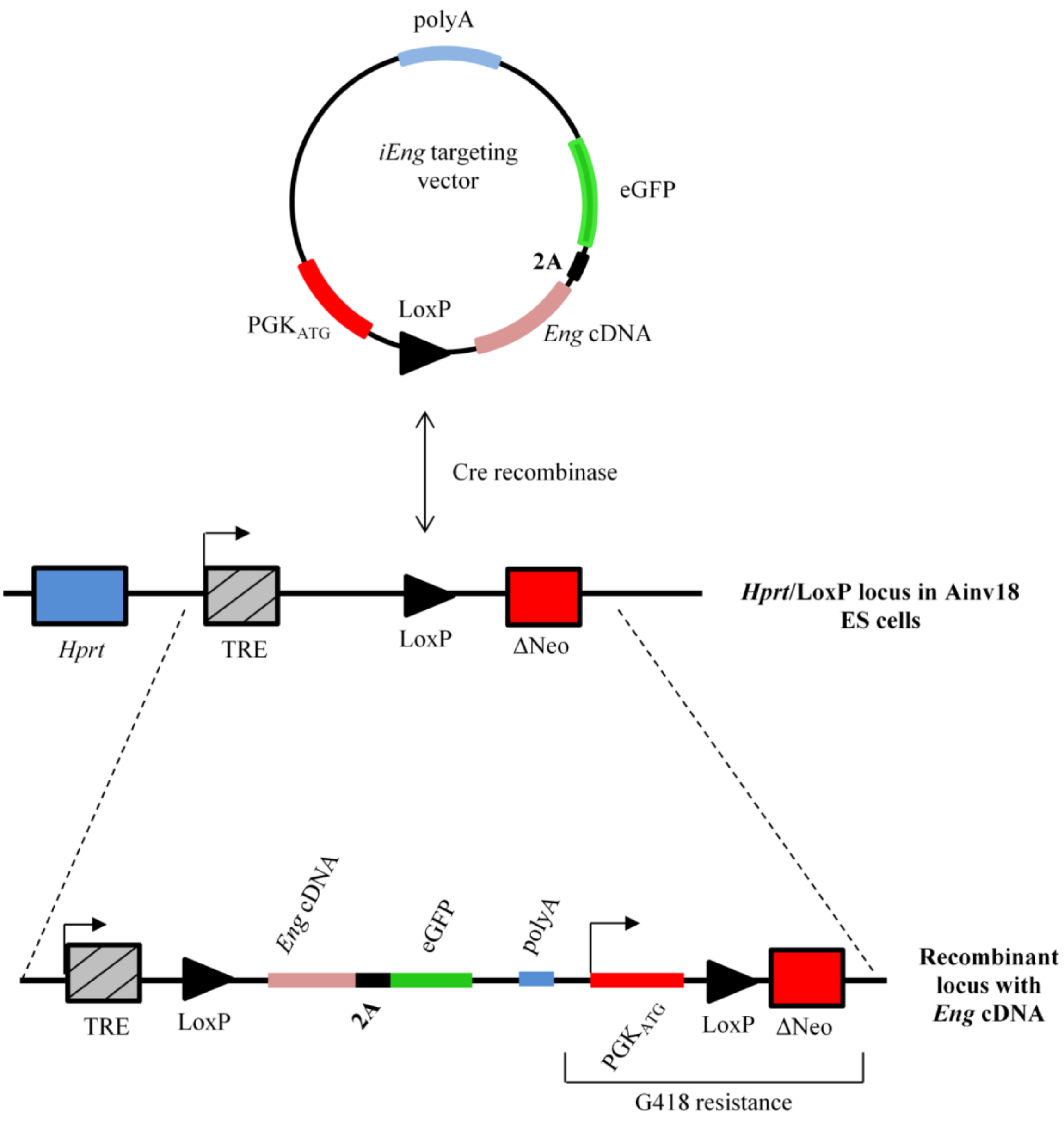


**Fig. S7. Schematic diagram of the strategy used to generate iEng:GFP embryonic stem cell line.** The construct containing Eng cDNA:2A-eGFP fragment was electroporated with a cre-recombinase expression plasmid into Ainv18 ES cells. Cre mediated recombination generated and ES cell line containing Eng cDNA:2A-eGFP fragment downstream of a tet responsive element (TRE). The reconstitution also generated a functional Neomycin resistance gene under PGKneo promoter, promoting clone selection using G418.

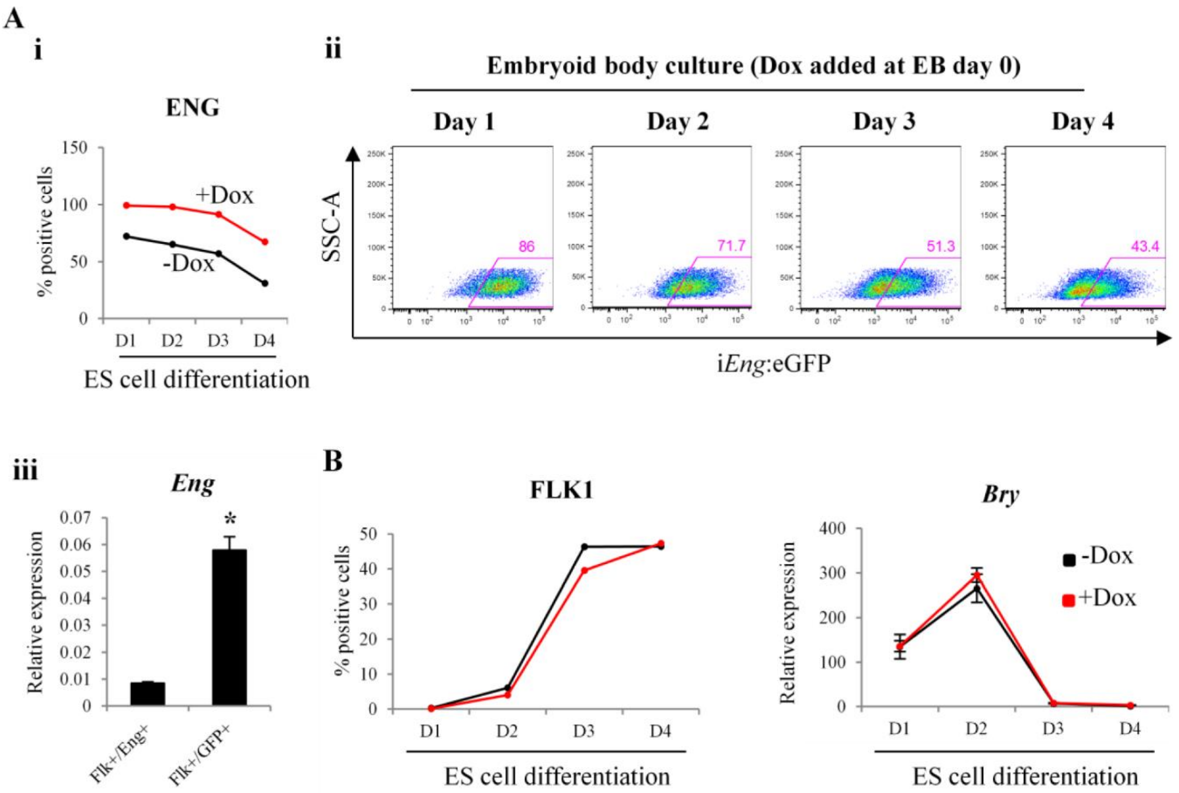


**Fig. S8. Dox induced expression of ENG in EBs.** (A) iEng:eGFP ES cells were cultured in the presence or absence of Dox for four days. Dox was added at the beginning of differentiation. (i) Histogram showing the percentage of ENG positive cells. (ii) Flow cytometry graphs showing the expression of GFP over 4 days of EB differentiation. (iii) RT-PCR showing the levels of *Eng* expression in $FLK1^+/ENG^+$ cells (Cells cultured without Dox) and $FLK1^+/GFP^+$ cells (Cells cultured in the presence of Dox) sorted from day 3 EBs. (B) Histograms showing the percentage of FLK1 positive cells and transcript abundance of Bry during days 1–4 of EB differentiation with and without Dox.*$P$<0.05.

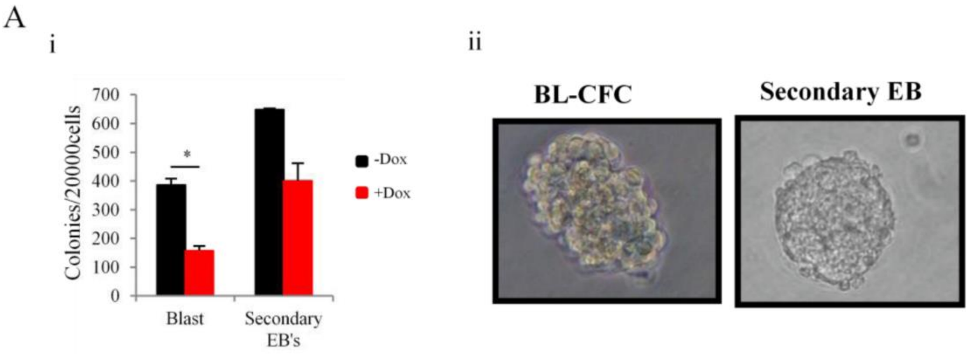


**Fig. S9. Endoglin overexpression early in ES/EB differentiation reduced BL-CFC numbers.** (A) (i) Bar chart showing the number of blast and secondary EB colonies. Cells were treated with or without Dox at the ES cell stage. Bulk day 2 EBs were dissociated and 20,000 cells were plated in 1 ml of BL-CFC mix. Blast colonies and secondary EBs were counted after 4 days of culture. The error bars represent standard deviation of the mean. The experiment was performed in technical triplicates. (ii) Phase contrast images showing a typical blast colony and secondary EB. Images were taken at 10×magnification. **P*<0.05.

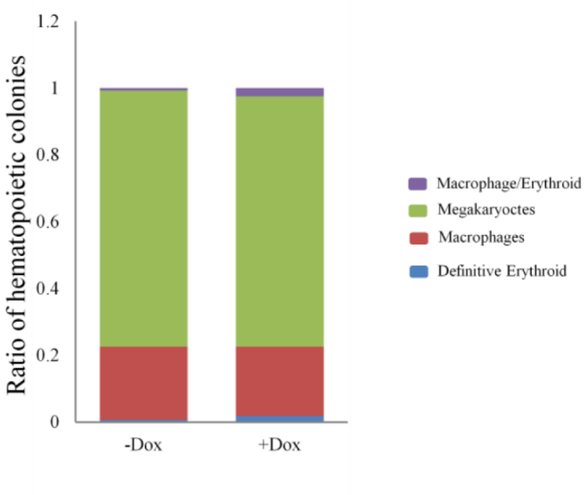


**Fig. S10. Ratios of definitive hematopoietic colonies generated by cells with forced ENG expression.**

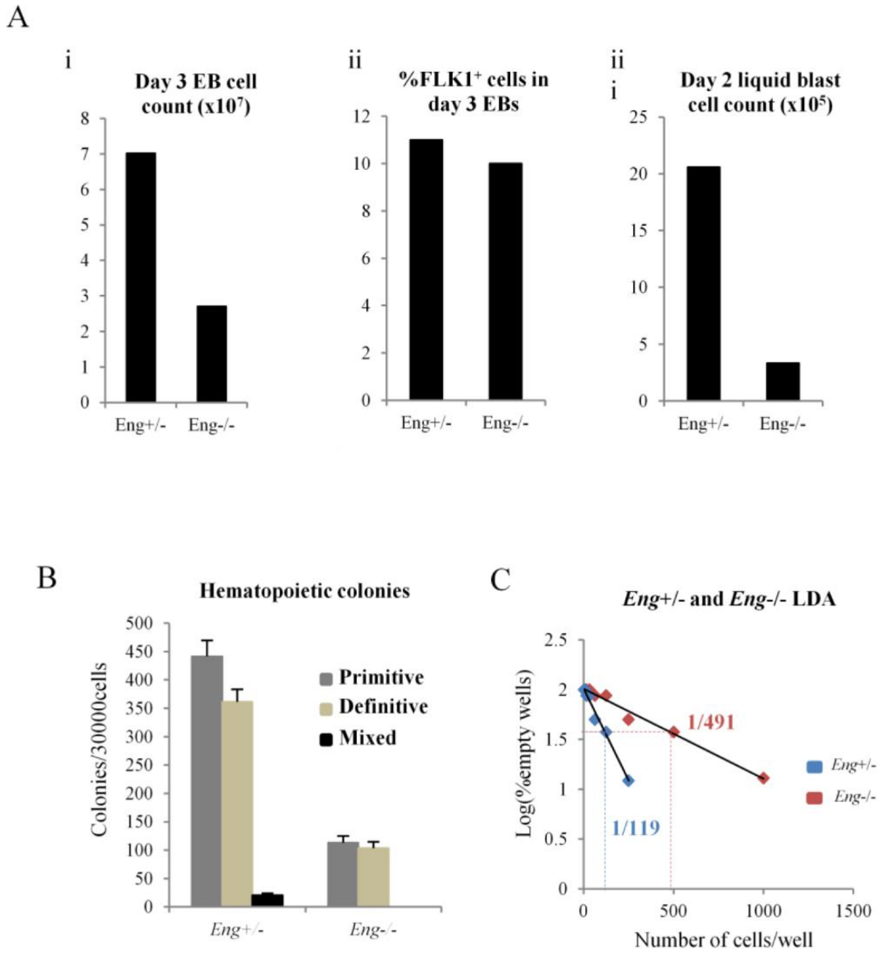


**Fig. S11. Comparative HE and hematopoietic potential of ENG$^{-/-}$ and ENG$^{+/-}$ ES cells.** (A) (i,ii) Bar chart showing detailed analysis of day 3 EBs derived from Eng$^{+/-}$ and Eng$^{-/-}$ ES cells. 3×10$^{4}$ ES cells from each cell line were seeded in EB media for 3 days followed by the quantification of (i) cell number and (ii) FLK1$^{+}$ cell percentage. (iii) Bar chart showing cell counts in day 2 liquid blast cultures. 8.5×10$^{4}$ FLK1$^{+}$ cells were sorted from day 3 EBs derived from Eng$^{+/-}$ and Eng$^{-/-}$ ES cells, then seeded in liquid blast cultures. After 2 days, the cells were collected and counted. (B) 3×10$^{4}$ cells collected from Eng$^{+/-}$ and Eng$^{-/-}$ day 2 liquid blast cultures were seeded in methylcellulose hematopoietic assays. Primitive and definitive colonies were counted after four and nine days respectively. (C) FLK1$^{+}$ cells collected from Eng$^{+/-}$ and Eng$^{-/-}$ day 3 EBs were seeded in LDA assays and wells were scored after four days. The data showed that the frequency of hematopoietic progenitors decreases from 1:119 (Eng$^{+/-}$ ES cells) to 1:491 (Eng$^{-/-}$).

**Table S1: Primary and secondary antibodies used for flow cytometry and cell sorting.**

| Antigen name | Isotype/conjugate | Clone | Company/catalogue number |
|---|---|---|---|
| TIE2 (CD202) | Rat/Biotin | TEK4 | ebioscience/13-5987 |
| | Rat/PE | TEK4 | ebioscience/12-5987 |
| Endoglin (CD105) | Rat/Biotin | MJ7/18 | ebioscience/13-1051 |
| | Rat/PE | MJ7/18 | ebioscience/12-1051 |
| | Rat/Pacific blue | MJ7/18 | Biolegend/120411 |
| c-KIT (CD117) | Rat/ APC | 2B8 | ebioscience/17-1171 |
| | Rat/eFluor780 | 2B8 | ebioscience/47-1171 |
| Flk1 (CD309) | Rat/Biotin | Avas12α1 | ebioscience/13-5821 |
| | Rat/APC | Avas12α1 | ebioscience/17-5821 |
| | Rat/PE | Avas12α1 | ebioscience/12-5821 |
| CD41 | Rat/Biotin | MWReg30 | ebioscience/13-0411 |
| | Rat/Pe-Cy7 | MWReg30 | ebioscience/25-0411 |
| | Rat/APC | MWReg30 | ebioscience/17-0411 |
| CD45 | Rat/percp-cy5.5 | 30-F11 | Biolegend/103132 |
